# Nonempirical Simulations of Inhomogeneous Broadening of Electronic Transitions in Solution: Predicting Band Shapes in One- and Two-Photon Absorption Spectra of Chalcones

**DOI:** 10.3390/molecules22101643

**Published:** 2017-09-30

**Authors:** Joanna Bednarska, Robert Zaleśny, Guangjun Tian, Natarajan Arul Murugan, Hans Ågren, Wojciech Bartkowiak

**Affiliations:** 1 Department of Physical and Quantum Chemistry, Faculty of Chemistry, Wrocław University of Science and Technology, Wyb. Wyspiańskiego 27, PL-50370 Wrocław, Poland; 2 Hebei Key Laboratory of Microstructural Material Physics, School of Science, Yanshan University, Qinhuangdao 066004, China; tian.phys@gmail.com; 3 Division of Theoretical Chemistry and Biology, School of Biotechnology, Royal Institute of Technology, SE–10691 Stockholm, Sweden; murugan@kth.se (N.A.M.); hagren@kth.se (H.A.)

**Keywords:** density functional theory, two-photon absorption, vibrationally-resolved spectra, hybrid QM/MM solvation models

## Abstract

We have examined several approaches relying on the Polarizable Embedding (PE) scheme to predict optical band shapes for two chalcone molecules in methanol solution. The PE-TDDFT and PERI-CC2 methods were combined with molecular dynamics simulations, where the solute geometry was kept either as rigid, flexible or partly-flexible (restrained) body. The first approach, termed RBMD-PE-TDDFT, was employed to estimate the inhomogeneous broadening for subsequent convolution with the vibrationally-resolved spectra of the molecule in solution determined quantum-mechanically (QM). As demonstrated, the RBMD-PE-TDDFT/QM-PCM approach delivers accurate band widths, also reproducing their correct asymmetric shapes. Further refinement can be obtained by the estimation of the inhomogeneous broadening using the RBMD-PERI-CC2 method. On the other hand, the remaining two approaches (FBMD-PE-TDDFT and ResBMD-PE-TDDFT), which lack quantum-mechanical treatment of molecular vibrations, lead to underestimated band widths. In this study, we also proposed a simple strategy regarding the rapid selection of the exchange-correlation functional for the simulations of vibrationally-resolved one- and two-photon absorption spectra based on two easy-to-compute metrics.

## 1. Introduction

**Figure 1:**
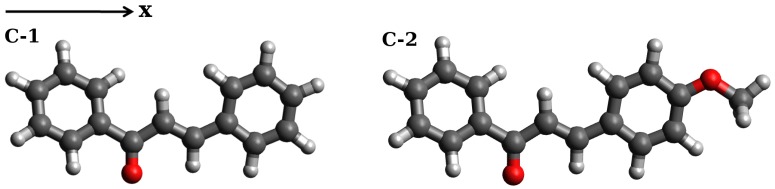
The structures of the **C-1** and **C-2** molecules studied in the present work. Shown is also their orientation in the Cartesian coordinate system adopted for second-order transition moment calculations (see the text for details).

During last three decades, we have witnessed a rapid development of electronic structure theories. These efforts encompass new methods, approximations and efficient algorithms; see Refs. [1,2,3] and the references therein. In particular, local approximations combined with ab initio methods have made it possible to study electronic excitations in the case of large molecules [2]. The result of this development is that fairly sophisticated methods nowadays can be applied to determine the electronic structure of molecules in the gas phase. Systems composed of dozens of atoms may be studied using Complete Active Space Self-Consistent Field (CASSCF) and Coupled-Cluster (CC) methods with basis sets of triple-ζ quality. For example, due to efficient implementations and the applications of the Resolution of Identity (RI) approximation [4], the optimization of excited-state geometries of medium-sized molecules in vacuo using the CC2 model is now a routine task. The progress in electronic structure theories has been paralleled by a development in solvation models [5,6,7,8,9,10,11,12,13,14,15]. Although some of these approaches retain a discrete solvent representation, still the most frequent choice to study the electronic structure of molecules in solution is to combine the continuum solvation model with the vertical approximation at the TDDFT level. On the one hand, this makes it possible to study molecules composed of more than hundreds of atoms but, on the other hand, such an approach lacks some important effects, e.g., the molecular vibrations or specific directional interactions with the solvent environment are not considered. It should not also be overlooked that the choice of approximate exchange-correlation functional is a pivotal step on the way to reliable simulations of electronic spectra [16,17,18,19,20,21,22,23,24,25]. Nowadays, numerous research groups aim at further refinements of the computational protocols for simulations of electronic One-Photon Absorption (1PA) and Two-Photon Absorption (2PA) spectra of molecules in solution. These efforts include explicit quantum-mechanical treatments of molecular vibrations (and also accounting for non-Condon effects) [26,27,28,29,30,31,32,33,34,35,36] and the nonempirical simulations of band shapes in the electronic one- and multi-photon spectra of solvated molecules [37,38,39,40,41]. These two subjects are also central to this study. The key ingredient in the computational protocol employed for the simulations of electronic one-photon absorption and two-photon absorption spectra of molecules in solution is the estimation of band broadening since it affects the absorption cross section. One of the approaches to determine the parameter in question is to either fit the line shape function to the experimental data or to use an arbitrary value, e.g., 0.1 eV. As a step towards the refinement of the protocol, herein we perform fully-nonempirical simulations including the determination of band broadening by using the hybrid Quantum Mechanics/Molecular Mechanics approach (QM/MM). For this purpose, we employed the polarizable embedding approach combined with TDDFT and also examined their performance by comparison with CC2 results. The choice of RI-CC2 as a reference method was dictated by its ability to deliver an accurate description of spectroscopic properties along with a reasonable computational cost for medium-sized systems and the availability of its efficient implementation combined with the polarizable embedding approach. In our recent paper, we demonstrated that a polarizable embedding solvent model, retaining a discrete representation of the environment, can be successfully combined with quantum-mechanical calculations of the vibrational fine structure of absorption bands in the electronic spectra, thus allowing for fully-nonempirical simulations [42]. The study in question, performed for the representative fluorescent difluoroborates, showed that this approach successfully reproduced the structured and structureless absorption bands in the spectra of the solvated dyes [42]. As demonstrated in a follow-up article, it can be extended also to the simulations of multiphoton spectra [43]. Until now, the approach in question has only been applied to molecules carrying the BF${}_{2}$ moiety in the following solvents: chloroform [42], dimethylformamide [42], dichloromethane [43] and acetonitrile [43]. The present work is a development of our previous efforts in assessing the protocols for the nonempirical simulations of absorption bands in the one- and multi-photon spectra of molecules in solution. To this end, we simulate the optical band shapes corresponding to the bright $\pi \to \pi^{*}$ transition to the second excited state for two chalcones (1,3-diaryl-2-propen-1-ones) in methanol solution using several approaches within the QM/MM framework. The molecules under investigation, shown in Figure 1, have recently been studied by Abegão and co-workers with a focus on their nonlinear optical properties [44].

This paper is organized as follows. Firstly, we propose how to choose the reliable Exchange-Correlation Functional (XCF) for the simulations of band shapes in the one- and two-photon absorption spectra of the studied chalcones based on the calculations in the gas phase. Secondly, we examine several QM/MM approaches for band widths to find the most suitable solvation model for the simulations in methanol. Next, we simulate the band shapes in the 1PA and 2PA spectra by using the best-suited inhomogeneous broadening. Finally, we analyze the importance of non-Condon effects in the simulations of two-photon spectra and compare the results with the experimental data.

## 2. Results and Discussion

### 2.1. Selection of the Exchange-Correlation Functional

Pinpointing the exchange-correlation functional suitable for the modeling of both the band shapes and the positions is by no means a trivial task [45]. To overcome this, we will follow below a simple procedure to select the reliable XCF for the simulations of the one- and two-photon absorption spectra based on the results of calculations in the gas phase. In our previous study [46], we showed that the vibrational reorganization energy:

(1) $$\lambda_{\mathrm{vib}}=\frac{1}{2}\sum_{j=1}^{3N-6}\omega_{j}{\left(\Delta_{j}^{k}\right)}^{2}$$

involving dimensionless normal-mode displacements ($\Delta_{j}^{k}$) and normal mode frequencies ($\omega_{j}$), can be used as a convenient metric for quantifying the reliability of density functionals in predicting the one-photon absorption band shapes. In Equation (1), the summation runs over all normal modes of vibrations for a nonlinear system composed of *N* atoms. Within the approximation used here (see ”Computational Details”), $\Delta_{j}^{k}$ is evaluated based on the $k^{th}$ excited-state energy gradient at the ground state equilibrium geometry for the $j^{th}$ normal mode:

(2) $$\Delta_{j}^{k}=-\frac{1}{\omega_{j}^{2}}\frac{\partial E_{k}}{\partial Q_{j}}$$

As demonstrated in Ref. [46], for a wide set of difluoroborates and a few other organic compounds, the accurate prediction of the vibrational reorganization energy leads to absorption band shapes that better fit to the selected reference (e.g., obtained using coupled-cluster theory) and thus, it can be used as an easy-to-compute metric. The XCF providing the best match with some reference can then be used in subsequent calculations using more refined vibronic and environmental models. Below, in Section 2.1.1, we will use this approach to choose a most-suited XCF for the simulations of 1PA spectra of the studied chalcones, while in Section 2.1.2 we will introduce an additional metric to rapidly assess the performance of XCFs in predicting vibronic 2PA spectra. The results of calculations obtained using the RI-CC2 method will serve as reference.

#### 2.1.1. Simulations of One-Photon Absorption Spectra in Vacuo

**Figure 2:**
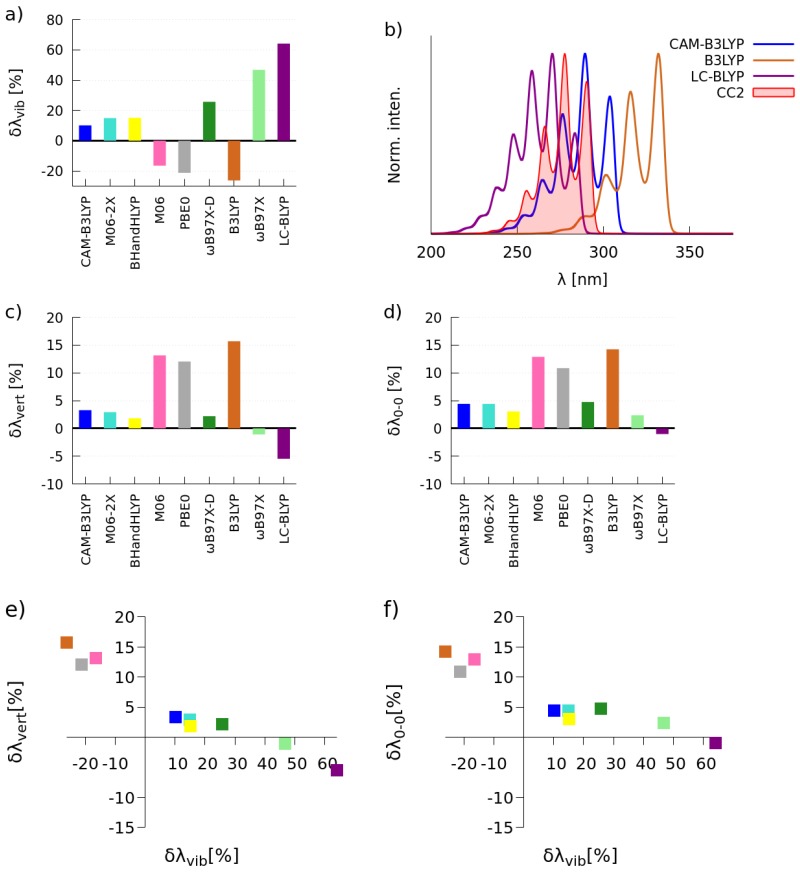
Bar charts showing the relative errors of $\lambda_{\mathrm{vib}}$ (**a**), $\lambda_{\mathrm{vert}}$ (**c**) and $\lambda_{0-0}$ (**d**) calculated with the selected XCFs with respect to the CC2 reference; the selected absorption spectra, see the text for details (**b**); graphs showing the relation of relative errors, i.e., $\lambda_{\mathrm{vert}}$ and $\lambda_{\mathrm{vib}}$ (**e**) and $\lambda_{0-0}$ and $\lambda_{\mathrm{vib}}$ (**f**) for the **C-1** molecule.

**Figure 3:**
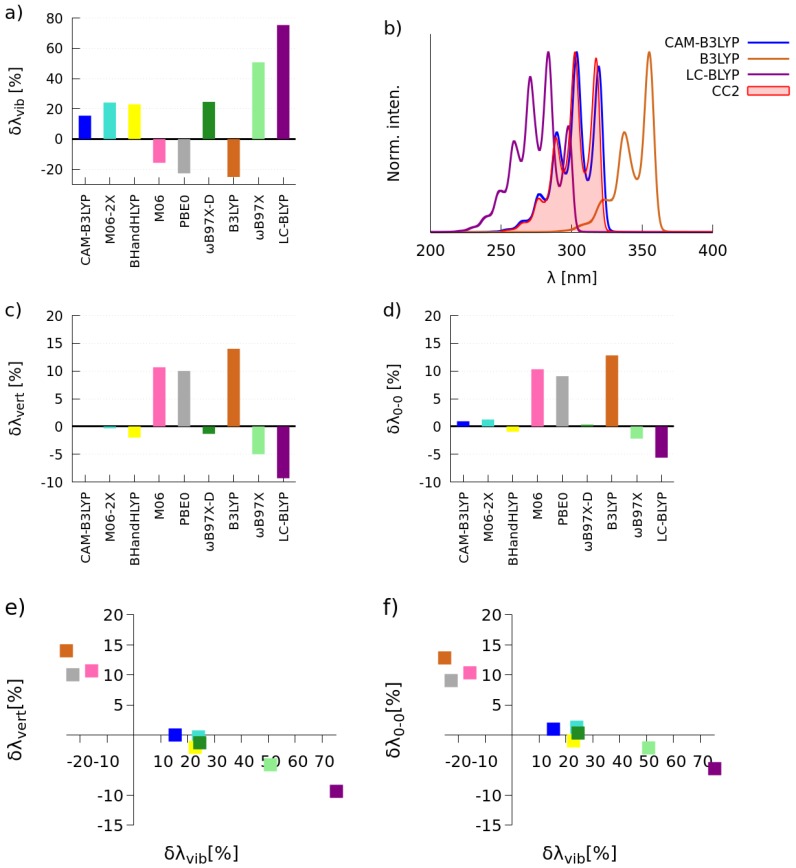
Bar charts showing the relative errors of $\lambda_{\mathrm{vib}}$ (**a**), $\lambda_{\mathrm{vert}}$ (**c**) and $\lambda_{0-0}$ (**d**) calculated with the selected XCF with respect to the CC2 reference; the selected absorption spectra, see the text for details (**b**); graphs showing the relation of relative errors, i.e., $\lambda_{\mathrm{vert}}$ and $\lambda_{\mathrm{vib}}$ (**e**) and $\lambda_{0-0}$ and $\lambda_{\mathrm{vib}}$ (**f**) for the **C-2** molecule.

The vibrational reorganization energies ($\lambda_{\mathrm{vib}}$), vertical excitation energies ($\lambda_{\mathrm{vert}}$) and 0-0 transition energies ($\lambda_{0-0}$) were calculated for the investigated **C-1** and **C-2** molecules in the gas phase by employing a wide palette of XCFs, i.e., B3LYP [47], PBE0 [48,49], M06 [50], BHandHLYP [51], CAM-B3LYP [52], M06-2X [50], ωB97X [53], ωB97X-D [54] and LC-BLYP [55]. The obtained values were compared with the results of calculations performed at the CC2 level of theory. The relative errors in $\lambda_{\mathrm{vib}}$, $\lambda_{\mathrm{vert}}$ and $\lambda_{0-0}$ with the tested XCFs with respect to the CC2 reference ($\delta \lambda_{\mathrm{vib}}=\frac{\lambda_{\mathrm{vib},\mathrm{TDDFT}}-\lambda_{\mathrm{vib},\mathrm{CC}2}}{\lambda_{\mathrm{vib},\mathrm{CC}2}}\times 100\%$) are shown in Figure 2 and Figure 3.

The Figure 2b depicts the simulated spectra by using the CC2 method and three XCFs, i.e., the two functionals that deliver the largest positive and negative relative errors and the one functional for which the smallest value of $\delta \lambda_{\mathrm{vib}}$ is found. For the sake of convenience of band shape comparisons, all presented spectra were normalized, i.e., the intensity of the highest peak was set to unity. As seen, the CAM-B3LYP, BHandHLYP, M06-2X and M06 functionals yield absolute relative errors smaller than 20% for molecule **C-1**. The largest positive and negative values of $\delta \lambda_{\mathrm{vib}}$ were obtained with LC-BLYP (64%) and B3LYP (−26%), correspondingly. As expected, the correct prediction of $\lambda_{\mathrm{vib}}$ is associated with the accurate reproduction of the vibrational fine structure of the absorption band, i.e., there is a similar intensity pattern for spectra marked in blue and red colors. In the case of calculations of vertical and 0-0 excitation energies, the CAM-B3LYP, M06-2X, BHandHLYP, ωB97X and ωB97X-D functionals deliver absolute relative errors less than 5%, whereas the largest positive error was obtained with B3LYP (around 15%). Analyzing simultaneously $\delta \lambda_{\mathrm{vert}}$ or $\delta \lambda_{0-0}$ and $\delta \lambda_{\mathrm{vib}}$ (see Figure 2e,f), it can be clearly seen that CAM-B3LYP, BHandHLYP and M06-2X are the best suited functionals for accurate simulations of absorption bands in the one-photon absorption spectra of the **C-1** molecule.

Figure 3 presents the same data, but for the **C-2** molecule. As one can notice, the CAM-B3LYP and M06 functionals deliver absolute relative errors smaller than 20%. In this case, M06-2X, BHandHLYP and M06 predict $\lambda_{\mathrm{vib}}$ with the error around 25%. Again, the largest positive error (75%) was predicted by LC-BLYP. As expected, the vibrational fine structure of the absorption band predicted by the best functional is close to the reference one, contrary to the results provided by LC-BLYP and B3LYP. In the case of the calculations of excitation energies, the CAM-B3LYP, M06-2X, BHandHLYP and ωB97X-D functionals yield results in satisfactory agreement with those predicted by the CC2 method ($\lambda_{\mathrm{vert}}$ and $\lambda_{0-0}$ less than 2%). Figure 3e,f show that CAM-B3LYP, BHandHLYP, M06-2X and ωB97X-D are quite accurate in reproducing the position and the shape of bands in the one-photon spectrum of the **C-2** molecule. Based on the above analysis, we have chosen the CAM-B3LYP functional for the subsequent simulations of one-photon spectra for **C-1** and **C-2** in methanol (see Section 2.2). Comparing Subfigure (e) in Figure 2 and Figure 3, one can note that the maximum of the absorption band for **C-2** is red-shifted with respect to the main spectral feature for **C-1**. This outcome parallels the experimental results obtained for compounds dissolved in methanol, i.e., the main spectral features for the **C-1** and **C-2** molecules were located at 308 nm and 343 nm, correspondingly. The bathochromic shift can be attributed to the electron-donating character of the methoxyl substituent in the aromatic ring of **C-2** that triggers a decrease in the energy gap [44].

#### 2.1.2. Simulations of Two-Photon Absorption Spectra In Vacuo

As shown in the preceding section, the most reliable functional, among those considered in this work, for predicting the shape and the position of the absorption band in the one-photon spectra of **C-1** and **C-2** is CAM-B3LYP. The simulations of two-photon absorption spectra, however, are more demanding both in terms of computational resources and the performance of the approximate XCFs. The latter aspect is demonstrated in Table 1, which contains a comparison of the two-photon strength ($\delta^{2\mathrm{PA}}$) for the first and second excited state for **C-1** and **C-2** calculated by employing the CC2 method and several XCFs. As seen, the first excited state exhibits negligible two-photon strength in comparison to the second excited state; hence, the transition to the second excited state is one- and two-photon allowed. Thus, simulated two-photon spectra presented in this study correspond to the $S_{2}\leftarrow S_{0}$ transition. Noteworthy, the majority of the employed functionals underestimate the values of $\delta^{2\mathrm{PA}}$ corresponding to the S${}_{2}\leftarrow$ S${}_{0}$ excitation when compared to CC2. This is in line with other studies on the performance of TDDFT in the calculations of the two-photon properties of representative medium-sized organic compounds and, most likely, emerges from the underestimation of the excited-state dipole moments [56,57]. Nevertheless, this issue has still not been satisfactorily addressed and demands further investigations.

**Figure 4:**
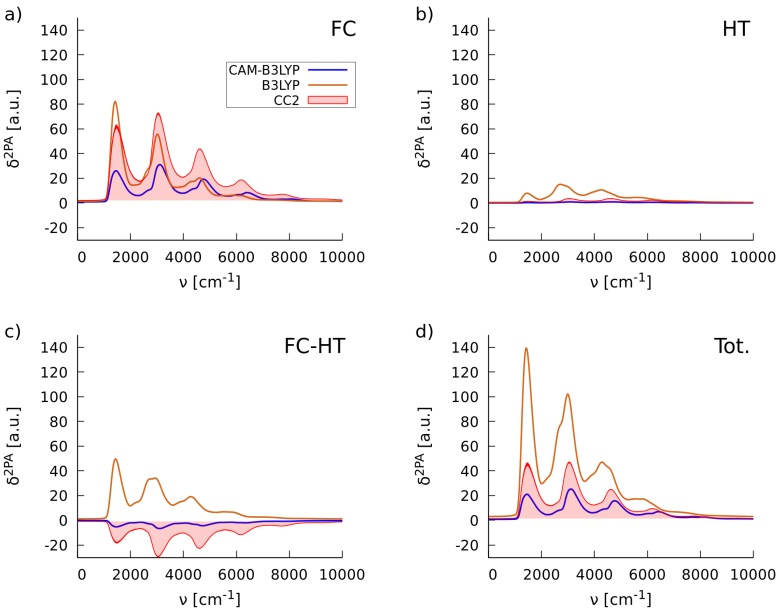
The simulated vibronic two-photon absorption spectra of the **C-1** molecule in vacuo with the B3LYP and CAM-B3LYP functionals and the CC2 method including the Franck–Condon (**a**), Herzberg-Teller (**b**) and Franck–Condon/Herzberg–Teller (**c**) contributions; and the total 2PA spectrum (**d**).

Yet another issue is the performance of density functional theory in predicting the vibronic profiles in two-photon absorption spectra. As shown by several works, even in the case of intense $\pi \to \pi^{⋆}$ excitations, the Franck–Condon approximation is often too crude for the simulations of the vibrational fine structure of absorption bands in two-photon spectra, thus one usually needs to include non-Condon effects [34,43,58,59,60,61]. This can be achieved, to a first approximation, by including the linear term in the expansion of the second-order transition moment with respect to the normal coordinates (see Equation (9)). Hence, due to the significance of non-Condon effects in the nonlinear absorption, the selection criteria of XCFs, outlined in the preceding section, might not be sufficient. In an attempt to adjust the procedure employed in Section 2.1.1 to the selection of XCFs for the simulations of vibronic 2PA spectra, we have introduced the additional guiding parameter:

(3) $${\left[S^{2}\right]}_{\alpha \alpha }=\sum_{j}\frac{\hbar }{2\omega_{j}}\frac{\partial S_{\alpha \alpha }}{\partial Q_{j}}\frac{\partial S_{\alpha \alpha }}{\partial Q_{j}}$$

which is the vibrational contribution to $\delta^{2\mathrm{PA}}$ within the harmonic approximation [62]; $S_{\alpha \alpha }$ stands for the second-order transition moment tensor and α∈{x,y,z}. To demonstrate the usefulness of this metric, we have calculated the three diagonal terms, i.e., ${\left[S^{2}\right]}_{xx}$, ${\left[S^{2}\right]}_{yy}$ and ${\left[S^{2}\right]}_{zz}$ for the **C-1** molecule in vacuo by using B3LYP and CAM-B3LYP functionals and the CC2 method. The results, listed in Table 2 show that the B3LYP functional, albeit the most accurate in predicting the electronic $\delta^{2\mathrm{PA}}$ value (cf. Table 1), delivers disastrous values of ${\left[S^{2}\right]}_{xx}$. This can be rationalized by the fact that the nuclear derivatives of second-order transition moments are overestimated to a large extent. On the other hand, the CAM-B3LYP functional is much more reliable in predicting ${\left[S^{2}\right]}_{xx}$ due to better agreement with the reference results. To demonstrate the differences in the predictions of vibronic two-photon absorption spectra by these two functionals, we present the corresponding spectra in Figure 4. As seen, the band shape predicted by the CAM-B3LYP functional is in a good agreement with the CC2 method. In conclusion, based on the results of the calculations in the gas phase presented in this section, we have chosen the CAM-B3LYP functional also for the simulations of band shapes in the two-photon absorption spectra of the chalcones in methanol solution.

### 2.2. Simulations of the One- and Two-Photon Spectra Including Solvation Effects

**Figure 5:**
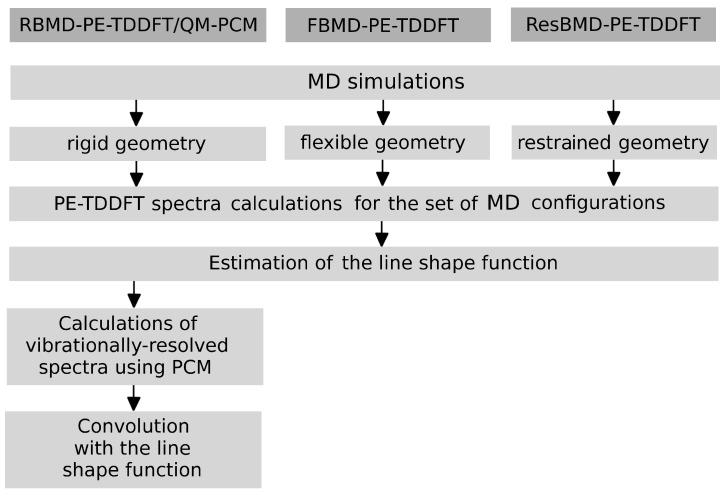
Schematic representation of the computational protocol used in this work to estimate the absorption band widths.

With the functional selected in the preceding paragraphs, we are now in position to estimate the absorption band widths for molecules in methanol solution. To this end, we have employed the QM/MM framework. In the first step of this procedure, the molecular dynamic simulations are performed to sample over solute-solvent configurations. In the second step, for the selected number of snapshots (see ”Computational details” for more information), the electronic structure calculations are carried out with the aid of TDDFT and CC2 methods combined with the polarizable embedding approach (PE) to represent the solvent environment. In this work, the sampling over solute-solvent configurations was performed in three variants, i.e., employing the rigid (Rigid Body (RB)), flexible (Flexible Body (FB)) and partly-flexible (Restrained Body (ResB)) representation of the solute geometry. In the first variant, the vibrational degrees of freedom of the solute are frozen, which allows for the estimation of the inhomogeneous broadening due to interactions with the environment. In this case, the simulation of absorption band shapes is then performed by convolution of the vibrationally-resolved spectra ($\sigma_{\mathrm{vib}}\left(\omega \right)$) with the normalized Gaussian function representing the inhomogeneous broadening (g(ω)):

(4) $$\sigma_{\mathrm{tot}}\left(\omega \right)=\int \sigma_{\mathrm{vib}}\left(\omega^{\prime }\right)g\left(\omega -\omega^{\prime }\right)d\omega^{\prime }$$

where $\sigma_{\mathrm{tot}}$ is the total one-photon absorption cross-section. The approach in question, denoted as RBMD-PE-TDDFT/QM-PCM (or RBMD-PERI-CC2/QM-PCM) is schematically presented in Figure 5. In the same figure, two other variants are also presented, namely FBMD and ResBMD, which are used to determine the histograms representing the population of solute configurations per excitation energy interval, which can be directly compared to the experimental spectra. The RBMD-PE-TDDFT/QM-PCM approach is well suited for the structured and structureless absorption band shapes of the rigid and semi-rigid molecules, while the latter two approaches (FBMD-PE-TDDFT and ResBMD-PE-TDDFT) are expected to be reasonable choices for the simulations of structureless absorption bands with large broadenings. In what follows, we will firstly discuss the results of the calculations of broadening parameters obtained by using the three aforementioned approaches. Then, we will move on to the results of the simulations of the one- and two-photon spectral profiles for the investigated molecules in the solution and compare them to the experimental data.

**Figure 6:**
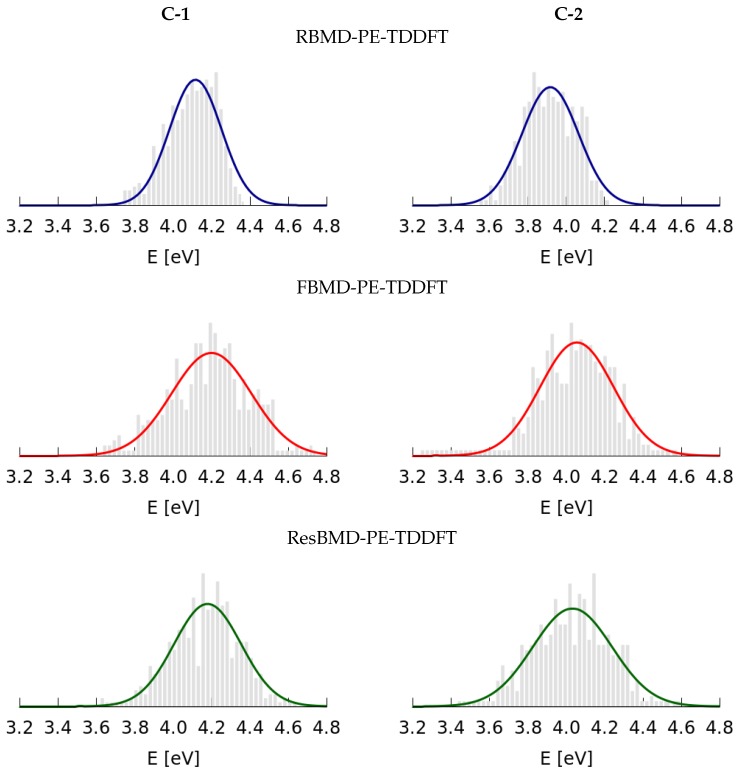
Histograms determined based on the results of calculations by using the RBMD-PE-TDDFT, FBMD-PE-TDDFT and ResBMD-PE-TDDFT approaches for uncorrelated **C-1** (**left**) **C-2** (**right**) configurations.

The results of calculations performed within the QM/MM framework are presented in Figure 6 and listed in Table 3. Figure 6 shows the histograms representing the population of conformations with respect to the transition energy to the S${}_{2}$ state. Table 3 contains the comparison of the results for **C-1** and **C-2** obtained by employing all examined approaches. As expected, the bands simulated by using the FBMD and ResBMD schemes have larger widths than those from the RBMD variant, hence they are characterized by larger values of standard deviation (σ), i.e., the difference is equal to 0.08 eV and 0.05 eV for FBMD and ResBMD, correspondingly for the molecule **C-1**. In this case, the FBMD simulations provide values of σ equal to 0.21 eV, whereas the calculations employing the ResBMD variant predict a broadening equal to 0.18 eV. This comes as no surprise, since in the FBMD approach, sampling over a larger conformational space is employed. It is noteworthy that the PE-TDDFT approach predicted band positions located at too high energy in the spectral region as compared to the experimental data. On the other hand, a bathochromic shift for **C-2** with respect to **C-1** was reproduced.

**Figure 7:**
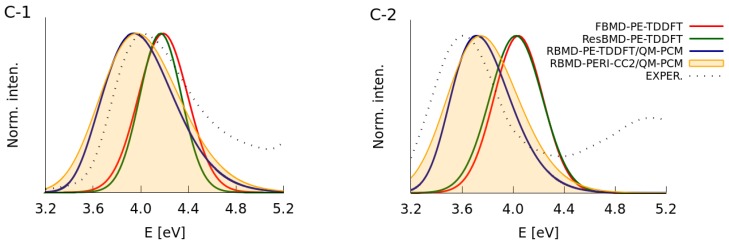
The comparison of the experimental spectra for the **C-1** and **C-2** molecules with the results of simulations employing nonempirical broadenings.

The RBMD simulations were combined with both the PE-TDDFT and PERI-CC2 methods. According to RBMD-PERI-CC2, the values of σ are equal to 0.20 eV and 0.21 eV for **C-1** and **C-2**, correspondingly. These outcomes vary from the RBMD-PE-TDDFT calculations, which provide the σ parameters equal to 0.13 eV and 0.15 eV. To choose a value better fitting the experimental data, we performed a convolution of the stick spectra for **C-1** and **C-2** with the Gaussian line-shape functions employing thus determined parameters (RBMD-PE-TDDFT/QM-PCM and RBMD-PERI-CC2/QM-PCM). A detailed analysis of data presented in Table 3 and Figure 7 reveals that the most accurate broadening parameters were obtained with the PERI-CC2 framework. Clearly, the RBMD-PERI-CC2/QM-PCM approach employing broadening equal to 0.20 eV and 0.21 eV correspondingly for **C-1** and **C-2** yields asymmetric bands showing the best match with the experimental data. It is notable that slightly larger values of σ, determined experimentally, can emerge from the overlap of the band corresponding to the transition to a higher excited state.

**Figure 8:**
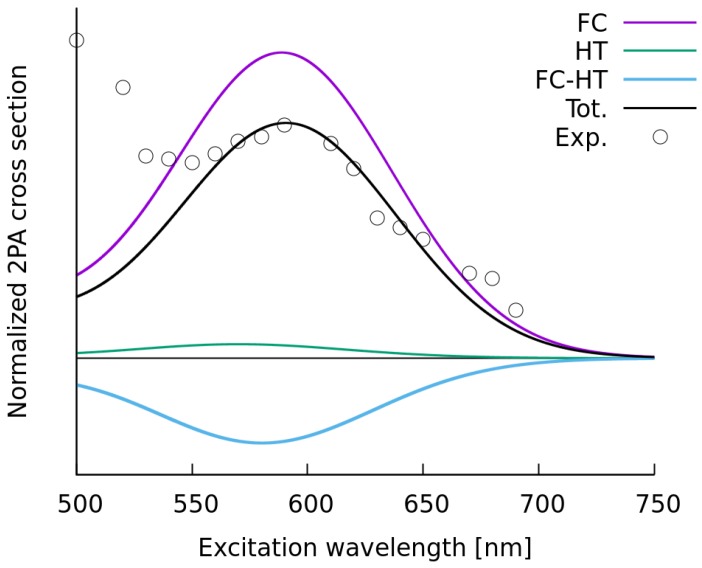
Comparison of the normalized experimental and the simulated two-photon absorption spectra of **C-1** in methanol.

The inhomogeneous broadening taken from the RBMD-PERI-CC2 calculations, represented by the Gaussian function with standard deviation equal to 0.20 eV, was then employed for the simulations of the two-photon spectra of the **C-1** molecule in methanol. To this end, we simulated the vibrationally-resolved two-photon spectra in methanol using the PCM solvation model and the formerly selected CAM-B3LYP functional and convoluted it with the line shape function including the thus determined broadening. In addition, we examined the importance of non-Condon effects, as it was performed for the simulations in vacuo. Figure 8 depicts the breakdown of total two-photon intensity into Franck–Condon (FC), Herzberg–Teller (HT) and the mixed Franck–Condon/Herzberg–Teller (FC/HT) terms. Based on this figure one can draw two conclusions. Firstly, the non-Condon effects make a substantial contribution to the total 2PA spectrum of **C-1** in methanol. Secondly, the protocol applied to account for vibronic effects and the nonempirical estimation of solvent effects leads to satisfactory agreement with the experimental band widths in the 2PA spectra (without taking into account the measurement uncertainty [44]).

## 3. Computational Details

### 3.1. Simulations of One-Photon Spectra

The calculations of one-photon spectra of **C-1** and **C-2** in the gas phase were performed at the DFT and CC2 levels of theory. The DFT calculations including the geometry optimization, the calculations of the ground-state Hessian and the excited-state energy gradient were performed with the GAUSSIAN 09 [63] package by employing nine exchange-correlation functionals combined with the cc-pVTZ basis set. All computations at the CC2 level of theory, aiming at predicting vibrational reorganization energy, were carried out with the aid of the TURBOMOLE package [64] using the the cc-pVDZ basis set. This choice was based on our previous studies showing that the differences in $\lambda_{\mathrm{vib}}$ with these two basis sets are insignificant [46]. To calculate the vibrationally-resolved optical spectra, we employed the linear coupling model [62,65]. This method assumes that the ground and excited-state energy surfaces are represented by displaced harmonic oscillators with unaltered curvatures. The results for the ground-state Hessian and excited-state energy gradient at the ground state equilibrium geometry were used to determine the dimensionless displacements (see Equation (2)). The transformation of excited-state gradients to the normal coordinate basis was done by using in-house computer routines. The vibrational fine structure of the one-photon band corresponding to transition to the second excited state was computed by using the wave packet approach with the aid of the orca_asa module as implemented in the ORCA package [66]. The corresponding spectra in gas phase were simulated assuming both homogeneous and inhomogeneous broadenings, represented by the Lorentzian (FWHM = 10 cm${}^{-1}$) and Gaussian (FWHM = 470 cm${}^{-1}$) functions, respectively. The simulations of the vibrationally-resolved spectra of **C-1** and **C-2** in methanol were carried out at the CAM-B3LYP/cc-pVTZ level of theory by employing a procedure similar to that used in the calculations in gas phase. The PCM solvation model was then employed to account for solvation effects [5].

### 3.2. Simulations of Two-Photon Spectra

The two-photon strengths of **C-1** and **C-2** were computed by employing quadratic response theory [67,68] combined with the TDDFT and CC2 methods. The rotationally-averaged two-photon strength ($\delta^{2\mathrm{PA}}$) is given by:

(5) $$\delta^{2\mathrm{PA}}=\frac{1}{30}\sum_{\alpha \beta }\left[2S_{\alpha \alpha }S_{\beta \beta }^{*}+2S_{\alpha \beta }S_{\alpha \beta }^{*}+2S_{\alpha \beta }S_{\beta \alpha }^{*}\right]$$

The second-order transition moment matrix elements ($S_{\alpha \beta }$) appearing in the above expression are defined within the DFT framework as [69,70]:

(6) $$S_{\alpha \beta }=\sum_{i}\left(\frac{\langle 0|\mu_{\alpha }\left|i\rangle \langle i\right|\mu_{\beta }|f\rangle }{\omega_{i}-\frac{\omega_{f}}{2}}+\frac{\langle 0|\mu_{\beta }\left|i\rangle \langle i\right|\mu_{\alpha }|f\rangle }{\omega_{i}-\frac{\omega_{f}}{2}}\right)$$

where 0, *i* and *f* stand for the ground, intermediate and final excited state; ℏω is the excitation energy to a given excited state. The two-photon absorption cross-section is defined based on the following formula:

(7) $$\sigma^{\left(2\right)}\left(\omega \right)=\frac{4\pi^{3}\alpha a_{0}^{5}\omega^{2}}{c}g\left(2\omega \right)\delta^{2\mathrm{PA}}$$

where g(2ω) is the line shape function, $a_{0}$ is the Bohr radius, α is the fine structure constant and *c* is the speed of light. In this study, the line shape function was represented by the Gaussian function:

(8) $$g\left(2\omega \right)=\frac{1}{\sigma \sqrt{2\pi }}\exp\left[-\frac{1}{2}{\left(\frac{2\omega -\omega_{f}}{\sigma }\right)}^{2}\right]$$

where σ is the standard deviation determined based on the results of the QM/MM simulations.

Vibrationally-resolved two-photon spectra calculations were performed including up to the linear term in the expansion of the second-order transition moment with respect to normal modes (*Q*):

(9) $$S_{\alpha \beta }\left(Q^{\prime }\right)\approx S_{\alpha \beta }\left(Q_{0}^{\prime }\right)+\sum_{j=1}^{3N-6}{\left(\frac{\partial S_{\alpha \beta }}{\partial Q_{j}^{\prime }}\right)}_{0}Q_{j}^{\prime }$$

where $Q^{\prime }$ is a normal mode of final state and the zero subscript corresponds to equilibrium geometry. It follows from the above equation that the simulations of vibrationally-resolved two-photon spectra require the calculations of nuclear derivatives of the second-order transition moment. For this purpose, numerical differentiation on the set of displaced geometries, with subsequent transformation to normal coordinate basis, was employed by using in-house computer routines. The stability of computations was controlled by the Rutishauser–Romberg algorithm [71] employing a set of atomic displacements according to the formula:

(10) $$\frac{\partial S_{\alpha \beta }}{\partial X_{n}}=\frac{S_{\alpha \beta }\left(X_{n}^{eq}+2^{k}\Delta \right)-S_{\alpha \beta }\left(X_{n}^{eq}-2^{k}\Delta \right)}{2^{k+1}\Delta }$$

where $X_{n}$ stands for the Cartesian coordinate basis. In these calculations, we used $\pm 2^{k}\Delta$, where *k* = 2 and Δ = 0.02 a.u. The vibrational fine structure of two-photon bands was performed using wave packet techniques with the aid of the DynaVib program [72].

### 3.3. Solvation Effects

The solvent-solute interactions were examined explicitly by employing the sequential QM/MM approach [41] and implicitly by using the PCM solvation model (in the case of the optimization of geometries and excited-state gradient calculations). The former approach involves the calculations of the optical properties as an average over uncorrelated configurations of **C-1** and **C-2** extracted from molecular dynamics (MD) trajectories, where the molecule under investigation was treated as a rigid, flexible or restrained body (with the force constant set to 100 kcal/mol). In other words, in the restrained body MD the molecular orientational and translational degrees of freedom are frozen for the molecule, but the vibrational motion is accounted for using harmonic potentials (which describe the motion of each atom around its equilibrium position). The MD simulations were carried out by using the AMBER 14 package [73]. The minimal energy structures of **C-1** and **C-2** were obtained at the B3LYP/6-311++G(d,p) level of theory employing the PCM solvation model with the aid of the GAUSSIAN 09 suite of programs [63]. The atomic charges (obtained by employing the CHELPG [74] procedure) and the generalized AMBER force field were employed to describe the electrostatic and van der Waals interactions of the investigated compound and the methanol molecules, with the cut off value equal to 12 Å. The simulation box for **C-1** contained 4641 methanol molecules with a dimension of approximately 73 × 66 × 63 Å${}^{3}$, whereas the cell lengths for **C-2** were assumed as follows: 75 × 66 × 63 Å${}^{3}$. In this case, the simulation box contained the **C-2** molecule surrounded with 4797 methanol molecules. The simulations were carried out within the isothermal-isobaric ensemble at room temperature and 1 atm pressure, employing a 2-fs time step to integrate the equation of motion. The total time scale for the production run was equal to 10 ns. To estimate the optical band widths, 500 solute configurations were extracted from the trajectories obtained in the course of RBMD, FBMD and ResBMD simulations and were used in the subsequent linear response calculations employing the polarizable embedding scheme (PE-TDDFT and PERI-CC2) [9,10,12]. The computations within PE-TDDFT framework were carried out with the aid of DALTON program [75], whereas the PERI-CC2 computations were conducted as implemented in the TURBOMOLE program [64]. At the final stage of this procedure, the Gaussian function was fitted to the normalized population of transition energies for the set of configurations exhibiting the bright transition to the second excited state.

## 4. Conclusions

Aiming at the accurate nonempirical simulations of the one- and two-photon absorption spectra of molecules in solution, we have in this study examined several approaches relying on the polarizable embedding scheme to predict optical band shapes for two chalcone molecules in methanol solution. To this end, we have combined the PE-TDDFT and PERI-CC2 methods with molecular dynamics simulations, where the solute geometry has been treated either as a rigid body, flexible or a partially-flexible body. The first approach, termed RBMD-PE-TDDFT, has been used to estimate the inhomogeneous broadening for subsequent convolution with the vibrationally-resolved spectra of the molecule in solution determined Quantum-Mechanically (QM). As demonstrated in this study, the RBMD-PE-TDDFT/QM-PCM approach delivers accurate band widths also reproducing the correct asymmetric shapes. Further refinement can be obtained by the estimation of the inhomogeneous broadening using the RBMD-PERI-CC2 method. On the other hand, the other two approaches (FBMD-PE-TDDFT and ResBMD-PE-TDDFT), which lack a quantum-mechanical treatment of the molecular vibrations, lead to underestimated band widths.

A simple strategy has also been proposed regarding the choice of exchange-correlation functionals for the simulations of one- and two-photon absorption spectra, including the vibrational fine structure of the absorption bands. We have here employed two easy-to-compute metrics, i.e. the vibrational reorganization energy and the harmonic vibrational contribution to the 2PA strength. Both are convenient metrics for the rapid selection of the most suited functional for the vibrationally-resolved 1PA and 2PA calculations. We have proposed that both parameters can be evaluated for molecules in the gas phase employing a medium-sized basis set. The functional best-matching the reference values of both metrics (e.g., obtained using the CC2 method) can then be used in subsequent calculations using more refined vibronic and environmental models.

## Figures and Tables

**Table 1:** Excitation energy (ΔE), the longitudinal element of second-order transition moment ($S_{xx}$) and the two-photon transition strength ($\langle \delta^{2\mathrm{PA}}\rangle$) for the two lowest-energy excited states of **C-1** and **C-2**. All calculations were performed with the cc-pVTZ basis set for geometries optimized at the B3LYP/6-311++G(d,p) level of theory. The orientation of molecules in the Cartesian coordinate system is shown in Figure 1.

	S${}_{1}\leftarrow$ S${}_{0}$			S${}_{2}\leftarrow$ S${}_{0}$		
	ΔE (eV)	$S_{xx}$ (au)	$\delta^{2\mathrm{PA}}$ (au)	ΔE (eV)	$S_{xx}$ (au)	$\delta^{2\mathrm{PA}}$ (au)
**C-1**						
BLYP	2.68	6	8	3.49	108	2.7 × 10${}^{3}$
PBE	2.64	5	6	3.51	107	2.6 × 10${}^{3}$
PBE0	3.30	9	14	4.01	111	2.7 × 10${}^{3}$
B3LYP	3.23	9	17	3.90	114	2.8 × 10${}^{3}$
CAM-B3LYP	3.57	7	11	4.28	93	1.8 × 10${}^{3}$
BHandHLYP	3.84	11	22	4.32	89	1.7 × 10${}^{3}$
CC2	3.43	6	6	4.47	137	4.0 × 10${}^{3}$
**C-2**						
BLYP	2.73	10	18	3.14	286	16.8 × 10${}^{3}$
PBE	2.70	9	14	3.15	289	17.2 × 10${}^{3}$
PBE0	3.34	12	27	3.72	221	10.2 × 10${}^{3}$
B3LYP	3.27	13	32	3.61	230	11.0 × 10${}^{3}$
CAM-B3LYP	3.60	9	17	4.04	171	6.1 × 10${}^{3}$
BHandHLYP	3.88	15	48	4.10	161	5.4 × 10${}^{3}$
CC2	3.45	9	17	4.07	270	15.5 × 10${}^{3}$

**Table 2:** Harmonic vibrational contribution to $\delta^{2\mathrm{PA}}$ for the S${}_{2}\leftarrow$ S${}_{0}$ transition. All values are given in au and were computed using the cc-pVTZ basis set.

B3LYP			CAM-B3LYP			CC2
${\left[S^{2}\right]}_{\mathrm{xx}}$	${\left[S^{2}\right]}_{\mathrm{yy}}$	${\left[S^{2}\right]}_{\mathrm{zz}}$	${\left[S^{2}\right]}_{\mathrm{xx}}$	${\left[S^{2}\right]}_{\mathrm{yy}}$	${\left[S^{2}\right]}_{\mathrm{zz}}$	${\left[S^{2}\right]}_{\mathrm{xx}}$
2342	2	<1	252	1	<1	356

**Table 3:** Summary of average excitation energies and broadening parameters represented by the Gaussian line shape function (σ is the standard deviation) for the **C-1** and **C-2** molecules.

	C-1		C-2	
	$E_{\max}$ (eV)	σ (eV)	$E_{\max}$ (eV)	σ (eV)
**PE-TDDFT**				
RBMD	4.09	0.13	3.91	0.15
FBMD	4.19	0.21	4.04	0.19
ResBMD	4.17	0.18	4.02	0.21
**PERI-CC2**				
RBMD	4.21	0.20	3.91	0.21
**PE-TDDFT/QM-PCM**				
RBMD	3.94	0.30	3.72	0.23
**PERI-CC2/QM-PCM**				
RBMD	3.94	0.33	3.72	0.28
**Experimental Data**				
	4.02	0.34	3.60	0.26

## Abbreviations

The following abbreviations are used in this manuscript:

TDDFT	Time-Dependent Density Functional Theory
1PA	One-Photon Absorption
2PA	Two-Photon Absorption
XCF	Exchange-Correlation Functional
PCM	Polarizable Continuum Model
RBMD	Rigid-Body Molecular Dynamics
ResBMD	Restrained-Body Molecular Dynamics
FBMD	Flexible-Body Molecular Dynamics
PE	Polarizable Embedding
RI	Resolution of Identity
QM	Quantum Mechanics
MM	Molecular Mechanics
